# DNA-PKcs, a player winding and dancing with RNA metabolism and diseases

**DOI:** 10.1186/s11658-025-00703-z

**Published:** 2025-03-04

**Authors:** Jiabao Hou, Mingjun Lu, Jingwei Guo, Jinghong Wu, Chenyang Wang, Ping-Kun Zhou, Teng Ma

**Affiliations:** 1 Cancer Research Center, Beijing Chest Hospital, Beijing Tuberculosis and Thoracic Tumor Research Institute, Capital Medical University, Beijing, 101149 China; 2 Beijing Key Laboratory for Radiobiology Beijing Institute of Radiation Medicine, Beijing, 100850 China

**Keywords:** DNA-PKcs, RNA metabolism, Transcriptional regulation, Alternative splicing, Noncoding RNAs

## Abstract

The DNA-dependent protein kinase catalytic subunit (DNA-PKcs) is a key kinase in the DNA repair process that responds to DNA damage caused by various factors and maintains genomic stability. However, DNA-PKcs is overexpressed in some solid tumors and is frequently associated with poor prognosis. DNA-PKcs was initially identified as a part of the transcription complex. In recent years, many studies have focused on its nonclassical functions, including transcriptional regulation, metabolism, innate immunity, and inflammatory response. Given the pleiotropic roles of DNA-PKcs in tumors, pharmacological inhibition of DNA-PK can exert antitumor effects and may serve as a potential target for tumor therapy in the future. This review summarizes several aspects of DNA-PKcs regulation of RNA metabolism, including its impact on transcriptional machinery, alternative splicing, and interaction with noncoding RNAs, and provides insights into DNA-PKcs beyond its DNA damage repair function.

## Introduction

The DNA-dependent protein kinase catalytic subunit (DNA-PKcs) is a serine/threonine protein kinase and a member of the phosphatidylinositol 3-kinase-related kinase (PIKK) family. DNA-PKcs has been broadly studied in the field of DNA damage repair, and it repairs broken DNA through nonhomologous DNA end-joining (NHEJ) to maintain genomic stability and integrity [1]. Briefly, the DNA-PK complex consists of Ku70, Ku80, and DNA-PKcs. When DNA double-strand breaks occur, Ku70/80 heterodimers recognize the DNA end and recruit DNA-PKcs to damaged sites, forming the DNA-PKcs-Ku-DNA complex [2]. Simultaneously, the serine/threonine protein kinase activity of DNA-PKcs is activated, which phosphorylates itself as well as a series of downstream proteins to repair damaged DNA. Dysregulation of DNA-PKcs expression and activity is usually tightly correlated with poor prognosis in patients with tumors. Therefore, DNA-PKcs are considered potential targets for tumor therapy [3]. Genetic and pharmacological methods to regulate DNA-PK function could affect downstream signal transduction and tumor proliferation as well as increase the radiosensitivity of tumor cells [4, 5].

Although the role of DNA-PKcs in DNA damage repair has been extensively studied, it was initially discovered as a part of the SP1 transcription complex [6]. The transcriptional regulation and gene expression status of cells changed when the inhibitor NU7441 was used. Subsequent studies revealed that DNA-PK plays an important role in transcriptional regulation. For example, DNA-PKcs alters the transcriptional activity and protein stability of some certain transcription factors and affects the transcription of downstream target genes. DNA-PKcs could also bind transcription factors, form transcription complexes, and regulate gene expression. In addition, some pathological factors, such as hypoxia and high NaCl concentration, can also induce transcriptional changes through DNA-PK [7, 8].

Compared with mRNA, noncoding RNAs such as long noncoding RNA (lncRNAs), micro RNA (miRNAs), and circular RNA (circRNAs) usually do not encode proteins. In recent years, researchers have found that noncoding RNA can affect the function of DNA-PKcs either by direct binding or by inhibiting its enzymatic activity. Moreover, DNA-PKcs influences ribosomogenesis as well as rRNA synthesis and translation. In addition to affecting mRNA transcription, DNA-PKcs also affects the alternative splicing of mRNA, which in turn affects the epigenetic regulation of genes, including *SRSF1* and *SRSF2* [9].

**Fig. 1:**
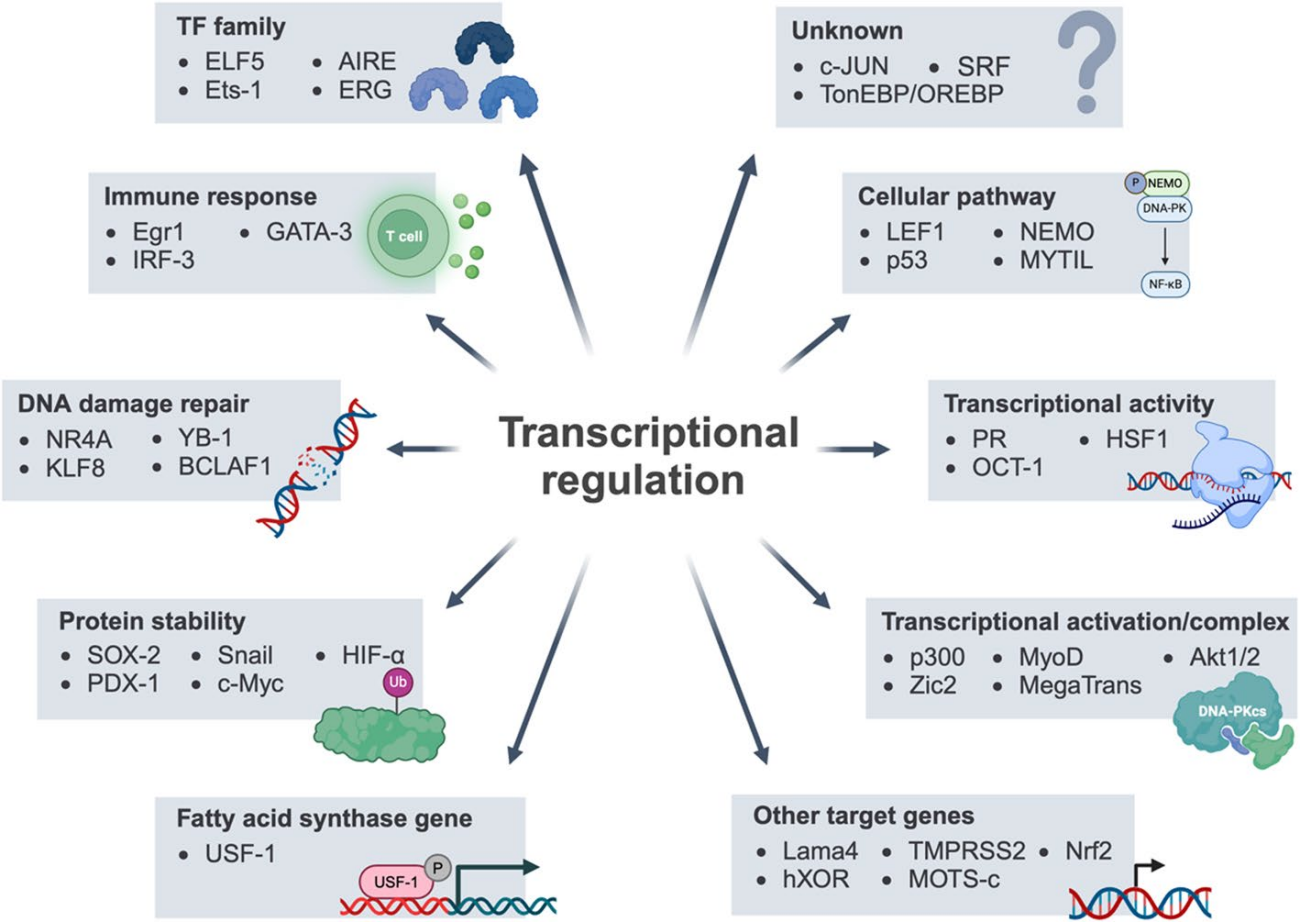
The pleiotropy of DNA-PKcs in transcriptional regulation. DNA-PKcs could affect gene transcription processes from different dimensions. It participates in basic transcription programs including transcription initiation, activation, and elongation. For some certain transcription factors, DNA-PKcs regulates transcriptional activity and protein stability of transcription factors to affect the function. In several transcription complexes, DNA-PKcs functions as an indispensable component. Moreover, DNA-PKcs is also involved in transcription of fatty acid synthase genes and regulating the differentiation of immune cells and the transcription of cytokine. In addition, DNA-PKcs could regulate DDR-related genes transcription processes in response to DNA damage. The figure was created with BioRender.com. P, phosphorylation; Ub, ubiquitination

In this review, we provide a detailed summary of DNA-PK functions in RNA metabolism, including transcriptional regulation (Fig. 1), noncoding RNA, and alternative splicing.

## DNA-PKcs in transcriptional regulation

DNA-PKcs was initially found as a part of transcriptional complex. Subsequently, researchers investigated global gene transcription changes in mammalian cells with DNA-PKcs inhibition or deficiency. It is reported that in vitro transcription levels of DNA-PK-deficient Chinese hamster ovary (CHO) cell extracts were reduced by twofold to sevenfold compared with the control cells [10]. Biological processes, including transcription and regulation of gene expression, changed after depletion of DNA-PKcs or treatment with inhibitor NU7441 [11]. Similarly, according to a recent study, the transcriptome changed after the treatment of NU7441 in human BT549 triple-negative breast cancer cells. RNA-seq results showed that there were significant differences in the genes of the pattern recognition receptor and cytoplasmic DNA-sensing pathways [12]. Although this indicates that DNA-PKcs may regulate global gene transcription, the mechanism has not been thoroughly explored.

The functions of DNA-PKcs on the transcription of certain genes including laminin alpha 4 (*Lama4*), NF-E2-related factor 2 (*Nrf2*), and human xanthine oxidoreductase gene (*hXOR*) were reported. Initially, F Bryntesson et al. found that *Lama4* gene transcription was regulated by DNA-PK through a radiation-independent manner in primary mouse embryo fibroblasts [13]. *Nrf2* is upregulated in various types of cancer and identified as a pharmacological target to overcome chemotherapy resistance. Wogonin could reverse resistance by inhibiting Nrf2/ARE pathway. DNA-PKcs are involved in wogonin-induced downregulation of Nrf2 mRNA at the transcriptional level in human myelogenous leukemia K562/A02 cells [14]. DNA-PKcs contributes to the regulation of hXOR mRNA expression induced by oncostatin M (OSM) [15]. Interestingly, DNA-PK could inhibit myelin transcription factor 1-like (MYT1L) mediated transcription in p53 mutated glioblastoma cells [16]. DNA-PK regulates Wnt signal transduction to control the metastatic phenotype in castration-resistant prostate cancer (CRPC) cells by interacting with the transcription factor LEF1. However, the molecular mechanism needs to be clearly elucidated [17].

It is mysterious to investigate how DNA-PKcs selectively regulate certain gene transcription or the global gene transcription.

### DNA-PKcs in transcription initiation, activation/repression, and elongation

Canonically, the global gene transcription program involves transcription initiation, activation, and elongation. DNA-PKcs acts as an additional component of the DNA topoisomerase IIβ (TopoIIβ) and poly[adenosine diphosphate (ADP)–ribose] polymerase–1 (PARP-1) complex to mediate transcription initiation [18, 19]. Consistently, other studies reported the presence of DNA-PKcs on the RNA polymerase II (Pol II) transcription units. DNA-PKcs is crucial for Pol II extension during transcriptional activation. Pause release, elongation of Pol II, and γH2AX levels were relatively reduced after using DNA-PKcs inhibitors [20, 21]. Furthermore, DNA-PK forms a complex with WW domain-containing protein 2 (WWP2) and RNA Pol II in response to DNA double-strand break (DSB). Proteasomes were recruited directly, leading to the degradation of RNA Pol II on damaged chromatin, thereby silencing the transcription of damaged genes [22]. DNA-PKcs can also remove RNA Pol II from DNA to induce transcriptional arrest in response to DSB [23]. Moreover, DNA-PKcs could prevent Ku heterodimers from overloading at a single DNA end, physically protecting transcription in the vicinity of DNA ends [24]. These findings suggest that DNA-PKcs may participate in transcription in the vicinity of DNA break sites.

Under most circumstances, DNA-PK regulates transcriptional activity of transcription factors through phosphorylation. Heat shock transcription factor 1 (HSF1) interacts with DNA-PK, and purified HSF1 stimulates DNA-PK phosphorylation in vitro. DNA-PK may also maintain the activated form of HSF1, thereby prolonging its transcriptional activity [25]. In addition, DNA-PK could phosphorylate the progesterone receptor (PR) in a DNA-dependent manner, and the inhibition of DNA-PK reduces the activation of RNA Pol II and the transcriptional activity of PR [26]. Moreover, octamer transcription factor-1 (Oct-1) was regarded as a stress sensor that promotes cell survival subsequent to DNA damage. DNA-PK could regulate the activity of the Oct-1 in response to DSB [27, 28]. However, the mechanism of action of the Q-rich domain of Oct-1 during this process is still unclear.

Hypoxia increases the stability and activity of the hypoxia-inducible factors (HIFs). DNA-PK assembles with hypoxia-inducible factor 1 (HIF-1) and tripartite motif containing protein 28 (TRIM28) to form heterotrimers, which stabilize the HIF occupancy of hypoxia response elements (HREs) and recruit cyclin-dependent kinase 9 (CDK9) to phosphorylate Pol II C-terminal domain (CTD) on Ser2, leading to HIF transcriptional activation in hypoxic breast cancer cells [7].

DNA-PK interacts with forkhead box A2 (FoxA2) and promotes its transcriptional activation by phosphorylating its Ser283 site [29]. It is recruited to the sites of AR action and initiates p300 occupancy, facilitating AR-dependent transcriptional transactivation [11].

DNA-PK affects transcription mediated by the Ets transcription factor family, which includes Ets-1, ERG, and ELF5. Ets-1 activates gene transcription involved in various cellular pathways, including proliferation, differentiation, adhesion, migration, and invasion. Its overexpression is associated with inflammation, invasive lesions, and cancer. The DNA-PK complex, as a partner of Ets-1, phosphorylates Ets-1 and regulates its transcriptional activity in a DNA-independent manner [30, 31]. Additionally, DNA-PKcs is a regulatory factor of ERG transcriptional activity, and ERG binds to DNA-PKcs at the Tyr373 site, inhibiting DNA-PKcs and altering the transcriptional activity of ETS target genes [32]. Moreover, the interaction between ELF5 and DNA-PKcs inhibits the activity of ELF5, thereby affecting partial transcription [33].

**Fig. 2:**
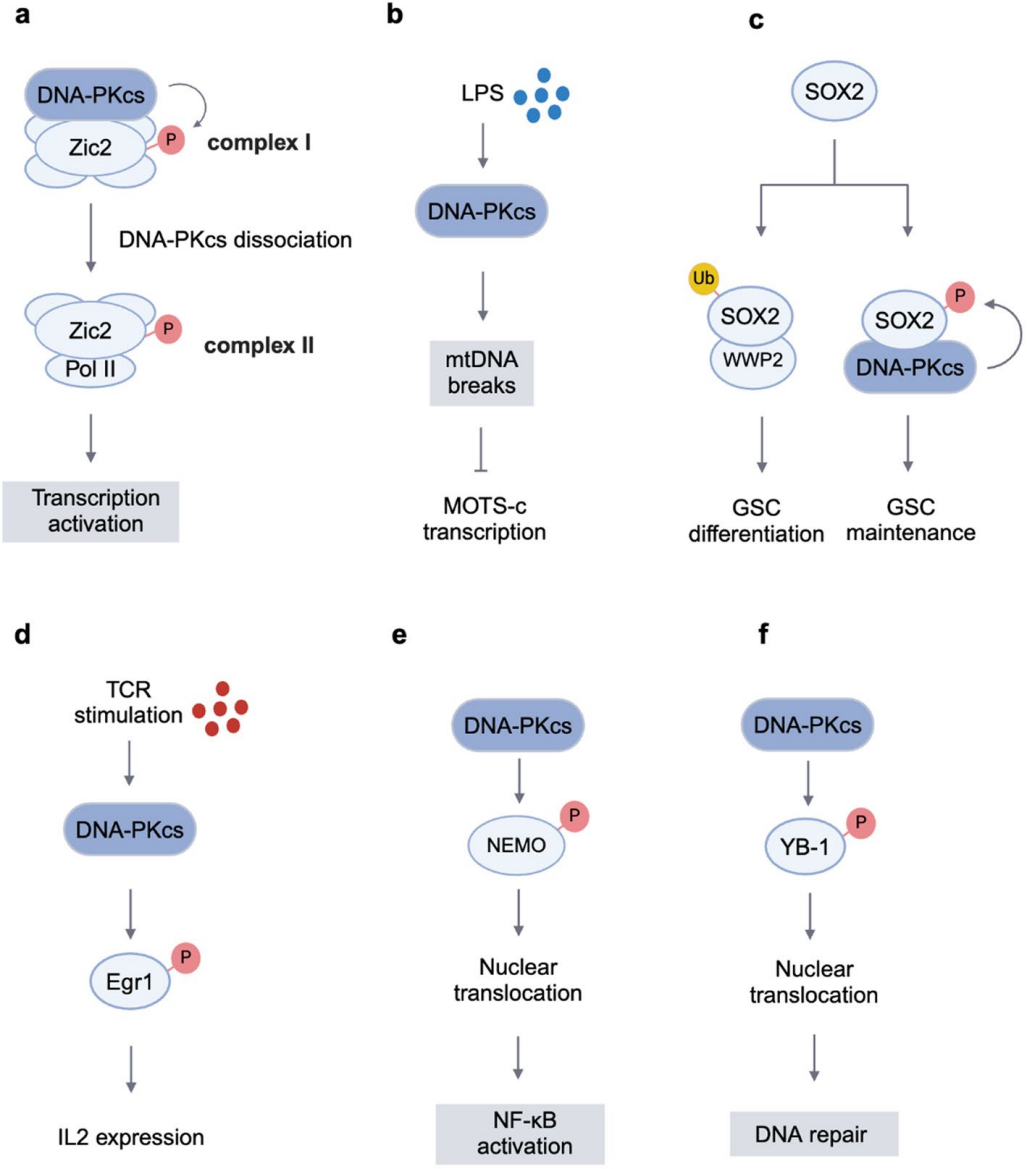
The mechanism of transcriptional regulation by DNA-PKcs. **a** DNA-PKcs could phosphorylate Zic2, and regulate the transformation of complex I to complex II. **b** DNA-PKcs induces mtDNA fragmentation and inhibits mitochondrial open-reading-frame of the 12S rRNA type-C (MOTS-c) transcription. **c** DNA-PKcs is an upstream regulator of SOX2 to stabilize the SOX2 protein and promote glioma stem cell maintenance. **d** DNA-PKcs phosphorylates Egr1, thereby promoting IL2 expression. **e** DNA-PKcs phosphorylates NEMO, promotes its nuclear translocation, and triggers NF-κB activation. **f** DNA-PKcs phosphorylates YB-1, promoting its nuclear translocation and enhancing DNA repair. The figure was created with BioRender.com. P, phosphorylation; Ub, ubiquitination

Zinc finger protein of cerebellum 2 (Zic2) is a transcription activator that plays a crucial role in forebrain development in mammals. The molecular mechanism of Zic regulation of transcription is mainly related to two types of complexes containing Zic2. As a component of the complex I, DNA-PKcs could phosphorylate Zic2, which is a necessary step for the transformation of complex I to complex II (Fig. 2a). It is also important for the interaction and subnuclear co-localization of Zic2 and RNA helicase A (RHA). Zic2 activates the transcription of target genes by binding to DNA and recruiting RHA. Transcriptional activation of Zic2 also depends on DNA-PK in vivo. Researchers found that Zic2 Ser200 may be the main phosphorylation site of DNA-PKcs. The interaction between Zic2 and RHA is reduced and transcriptional activation was significantly reduced when the mutation occurred [34, 35]. However, the factors affecting the disruption of complex II and the dephosphorylation of Zic2 are still unknown.

In insulin signal pathway, DNA-PK phosphorylates the Ser262 site of upstream stimulatory factor 1(USF-1) and then binds to the promoter region of fatty acid synthase (FAS), mediating the transcriptional activation of lipogenesis under feeding/insulin treatment [36]. USF is necessary for regulating FAS promoter activity during fasting/feeding. This study elucidated the relationship between DNA-PKcs and the insulin signal pathway. However, the interaction between transcriptional activation during the feeding process and the component proteins involved in DDR in the promoter region of adipogenic genes requires further exploration.

Cryptochrome (CRY) is a key component of the transcriptional repression complex and is involved in the regulation of the mammalian circadian clock. DNA-PK may indirectly and negatively regulate the phosphorylation of Ser588 at the C-terminus of CRY1. Loss or inhibition of DNA-PK leads to long-period rhythms [37].

DNA-PK could phosphorylate Ser435 and Ser446 of human serum response factor (SRF) [38]. The c-Jun Ser249 site is phosphorylated by DNA-PK in vitro, but the phosphorylation does not interfere with DNA binding [39].

In addition to affecting the phosphorylation of transcription factors, DNA-PKcs is also indispensable in several transcription complexes. The MEGATrans complex is necessary for activating enhancer RNA transcription and recruiting coactivators. It serves as a platform for recruiting specific enzymes (such as DNA-PK) to exert their effects. It serves as a hallmark that distinguishes the most active enhancers of the estrogen-regulated transcriptional program. Knockdown of DNA-PKcs or treatment with NU7441 significantly inhibited the activation of the estrogen receptor α-binding enhancer and its target genes expression [40]. The transcription factor tonicity-responsive enhancer/osmotic response element-binding protein (TonEBP/OREBP) can be activated by high NaCl concentrations, thereby increasing the transcription of protective genes. The DNA-PK subunit exists together with TonEBP/OREBP in the protein complexes formed in vitro. However, it is not clear whether it belongs to a part of the intracellular complex [8]. Moreover, DNA-PKcs interacts with other complexes, including transcriptional activator p300, myogenic determination factor MyoD, Akt1, and Akt2, to activate the transcription of the differentiation factor myogenin at its promoter without inducing DNA damage and repair [41].

Interestingly, DNA-PKcs induces mtDNA fragmentation through pathological mitochondrial division, inhibits MOTS-c transcription, and causes cardiac microcirculation dysfunction [42] (Fig. 2b). This suggests that DNA-PKcs may play a greater role in the transcriptional regulation of organelle genes.

### DNA-PKcs in regulation of transcription factors protein stability

In addition to the impact on transcriptional activity, previous studies have also investigated the role of DNA-PK in the stability of transcription factor proteins. DNA-PK phosphorylates pancreatic duodenal homeobox-1 protein (PDX-1) at the Thr11 site in response to DNA damage in mouse pancreatic beta cells. Moreover, PDX-1 is further degraded by proteasomes to reduce the expression of GLUT2 and glucokinase genes [43]. Hypoxia mainly induces changes in the higher-order chromatin structure, initiating DNA-PK activation in the absence of DNA lesions. Furthermore, DNA-PK is activated and protects HIF-1α from degradation, contributing to the expression of GLUT1 and adaptation to hypoxia [44]. However, DNA DSB also partially contributes to DNA-PK activation.

Moreover, DNA-PK promotes the malignant phenotype of tumors. Snail1 is a key regulator of epithelial-mesenchymal transition (EMT). The direct interaction between DNA-PKcs and Snail1 induces Snail1 phosphorylation at Ser100, increasing the stability of the Snail1 protein and potentiating Snail1 functions. Ultimately, it promotes the invasive ability of the tumor [45]. However, phosphorylated Snail1 exhibits feedback inhibition of DNA-PK kinase activity, hindering DNA damage repair and ultimately inducing genomic instability [45]. This indicates the feedback mechanism of substrates on DNA-PKcs. Cellular myelocytomatosis oncogene (c-Myc) is dysregulated in various human cancers. DNA-PKcs regulates the phosphorylation level of c-Myc at Thr58 through the Akt/GSK3 β pathway in human cervix cancer HeLa cells. Overexpression of DNA-PKcs induces increased protein stability of c-Myc, thereby promoting cell proliferation and carcinogenic transformation [46]. Fang et al. reported that DNA-PK is an upstream regulator of SOX2, which is a core transcription factor that maintains the properties of stem cells. It phosphorylates the Ser251 of human SOX2 to prevent WWP2-mediated ubiquitination, thereby stabilizing the SOX2 protein and promoting glioma stem cell (GSC) maintenance under normal circumstances. However, DNA-PK is disassociated from SOX2, which facilitates SOX2 binding to WWP2 and promotes SOX2 ubiquitination and GSC differentiation in response to DNA damage [47] (Fig. 2c). This reveals the function of DNA-PK in maintaining GSCs by precisely regulating the stability of the SOX2 protein in the presence or absence of DNA damage.

### DNA-PKcs in immune-related genes transcription

DNA-PKcs also participates in regulating the differentiation of immune cells and the transcription of cytokine. Research reported that DNA-PK regulated the differentiation of mouse CD4^+^ T cells into Th2 and Th1 cells by affecting the expression of GATA-3. Inhibition of DNA-PK through pharmacology or gene heterozygosity completely blocks the differentiation of CD4^+^ T cells into Th2 cells, as well as the expression of interleukin (IL)-5 and IL-13 [48]. DNA-PKcs plays a crucial role in the transcription of related genes after T cell receptor (TCR) stimulation. The Ser301 site of early growth response protein 1 (Egr1) is phosphorylated by DNA-PKcs in vitro. Inhibiting DNA-PKcs kinase activity reduces Egr1 protein expression by affecting protein stability, leading to a decrease in IL-2 level (Fig. 2d). Interestingly, phosphorylation of serine 301 of Egr1 is required for protein stability [49]. These studies provide critical targets for novel therapy for immune-related diseases. In addition, this also helps to understand the function of DNA-PKcs in immune cells.

DNA-PK acts as a DNA sensor upstream of the interferon (IFN) regulatory factor 3 (IRF-3) dependent innate immune response, triggering the transcription of type I interferon (IFN), cytokines, and chemokine genes [50]. When viral infection occurs, DNA-PK is activated to bind and phosphorylate the Thr135 site of IRF-3, reducing protein degradation caused by IRF-3 translocation to the cytoplasm and prolonging the half-life of IRF-3 [51].

The autoimmune regulatory factor (AIRE) is also regulated by DNA-PK. AIRE protein is a transcription factor expressed in myeloid thymic epithelial cells (mTECs) that plays a key role in central T cell tolerance. DNA-PK is involved in the phosphorylation of AIRE at Thr68 and Ser156, which influences its transactivation function [52]. In mice, DNA-PK is the direct molecular partner of AIRE, which cooperates with AIRE to induce peripheral tissue antigen transcription in mTECs [53]. In addition, mutated DNA-PKcs lead to impaired AIRE transcriptional activity in wild-type human fibroblasts, and DNA-PKcs may interact with AIRE to promote the expression of Toll-like receptor (TLR) 1, TLR3, and TLR8 in mouse RAW264.7 cells [54, 55].

Nuclear factor-kappa B (NF-κB) is a key transcription factor involved in immunity, inflammation, and cell transformation. Genetically toxic stress triggers phosphorylation of NF-κB essential modulator (NEMO) on the Ser43 site through the interaction between DNA-PK and NF-κB p65 subunit, allowing NEMO to shuttle through the nucleus and subsequently activate NF-κB (Fig. 2e). Moreover, DNA-PK could phosphorylate p50 subunit, promoting the stability of p50/p50 or p50/p65 dimers and their binding to promoters after tumor necrosis factor alpha treatment [56–58].

### DNA-PKcs in DDR related genes transcription

DNA-PK regulates many non-DNA damage repair (DDR) related genes transcription processes. However, owing to extensive research on its classical functions, many studies have reported that DNA-PK regulates transcription to promote DDR response. For example, DNA-PK directly phosphorylates the transcriptional regulator Y-box binding protein (YB-1) at Thr89, resulting in nuclear translocation, enhanced activity, and increased cellular DNA repair level [59]. However, the mechanisms involved in the intracellular signal pathway after YB-1 nuclear translocation are still unclear (Fig. 2f). In addition, DNA-PKcs drives Bcl-2-associated transcription factor 1 (BCLAF1) to the nuclear membrane by directly phosphorylating the key BCLAF1 RS residues, thereby regulating DNA damage repair. The knockdown of BCLAF1 also leads to weakened binding between Ku70 and DNA-PKcs [60]. Moreover, phosphorylation of Krüppel-like factor 8 (KLF8) at the Ser80 site by DNA-PKcs is necessary for DNA damage [61]. Nuclear receptor subfamily 4 group A (NR4A) is an orphan member of the nuclear receptor family that can function as a ligand-independent transcription factor. It interacts with DNA-PKcs and is recruited to repair lesions after DNA damage. DNA-PK mediates NR4A phosphorylation, thereby regulating DSB repair in mammalian cells. Interestingly, NR4A transcriptional activity is not necessary [62].

### To be investigated

Above all, the transcriptional regulation activity of DNA-PKcs largely depends on its kinase activity as the DNA-PK holoenzyme phosphorylation on other transcription machinery factors. The downstream mechanisms either involve conformational change or protein ubiquitination. However, the underlying mechanism remains unknown. It is necessary to use multi-omics approaches to identify the different binding/phosphorylated transcription factors for DNA-PK under different conditions, such as different organ development, disease progression, and subcellular organelle transcriptional regulation. Moreover, it is also important to discriminate the organization of DNA-PKcs/Ku70/Ku80 complex in DNA damage repair and transcription. Compared with the increasing resolution structures of DNA-PK complex in DNA damage repair, little is known about the DNA-PK transcription machinery. It is unknown how much Ku70/Ku80 contribute to the DNA-PKcs dependent transcription. Therefore, it is interesting to discriminate the Ku70/Ku80-dependent and Ku70/Ku80-independent roles in DNA-PKcs mediated transcription.

## DNA-PKcs in noncoding RNAs interaction network

DNA-PK can interact with various noncoding RNAs, including long noncoding RNA (lncRNAs), micro RNA (miRNAs), circular RNA (circRNAs), and small Cajal body-associated RNA (scaRNAs).

### LncRNAs

**Fig. 3:**
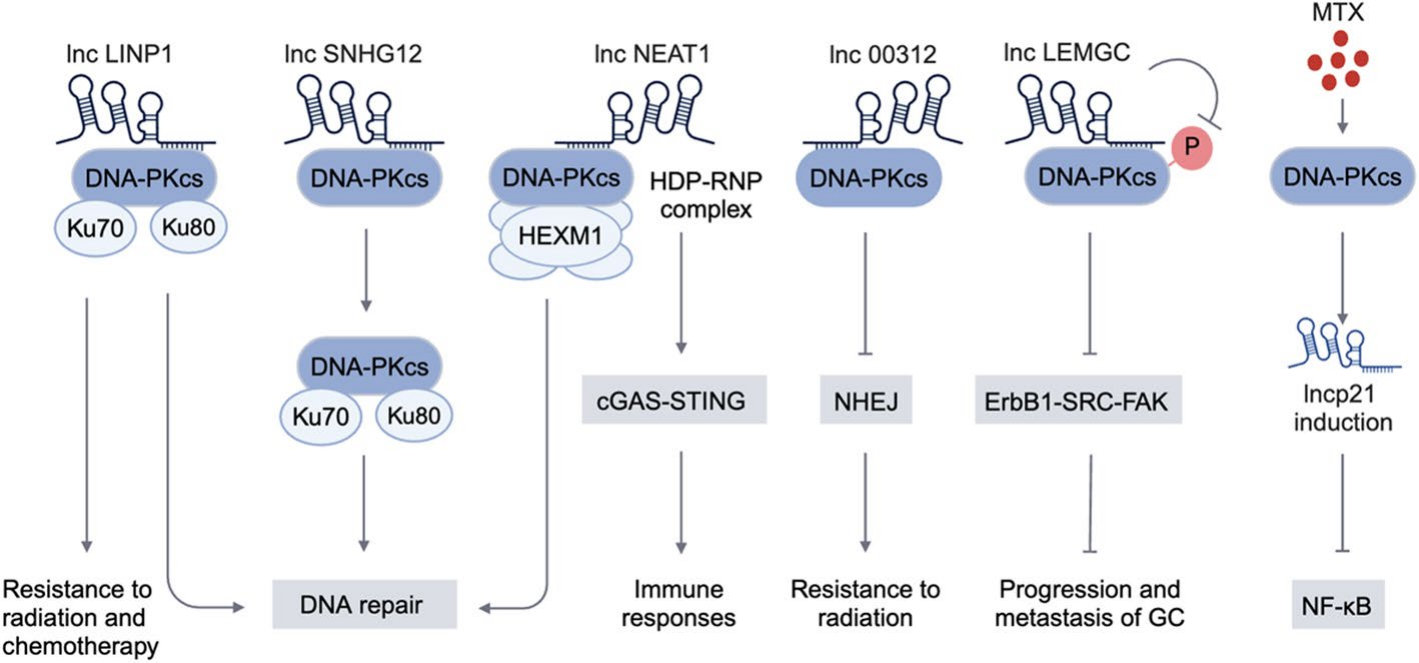
The interaction between DNA-PKcs and lncRNAs. The interaction between DNA-PKcs and lncRNA affects DNA repair, chemoradiotherapy resistance, and tumor progression. The figure was created with BioRender.com. P, phosphorylation

LncSNHG12 is a highly conserved lncRNA that can bind to DNA-PK, thereby promoting the ability of DNA-PKcs to bind to Ku70/Ku80 and regulate vascular DNA damage and cellular aging [63]. Lnc00312 directly binds to DNA-PKcs, obstructing the formation of the DNA-PK complex and inhibiting the NHEJ repair pathway, thereby enhancing the radiosensitivity of nasopharyngeal carcinoma cells [64]. Similarly, lncLINP1 and lncNEAT1 could also regulate DDR response through DNA-PKcs. LncLINP1 acts as a scaffold to interact with DNA-PKcs in response to DNA damage. Furthermore, knocking down LINP1 enhances the activation of radiation-induced apoptosis in HeLa cells [65]. LncNEAT1 is the core structural component of the nuclear paraspeckle (PS) organelles and it regulates DNA damage repair through ATM and DNA-PKcs [66]. In addition, HEXIM1 binds to lncNEAT1 to recruit the DNA-PK complex and paraspeckle proteins [67]. LncLINP1 binds to Ku80 and DNA-PKcs in the 5′ region (nucleotides 1–300) and 3′ region (nucleotides 600–917) of the transcript, enhancing DSB repair through the NHEJ pathway and promoting the resistance of triple-negative breast cancer cells to radiation and chemotherapy [68]. LncLEMGC interacts with DNA-PKcs and inhibits the phosphorylation of DNA-PKcs at Ser2056, ultimately inhibiting the progression and metastasis of gastric cancer by downregulating the ErbB1-SRC-FAK signaling pathway [69]. Research reported that methotrexate (MTX) activated DNA-PKcs in Jurkat cells and T cells, leading to the induction of TP53 and lncp21. Ultimately, lncp21 bound to RELA mRNA and inhibited NF-κB activity [70] (Fig. 3).

### CircRNAs

**Fig. 4:**
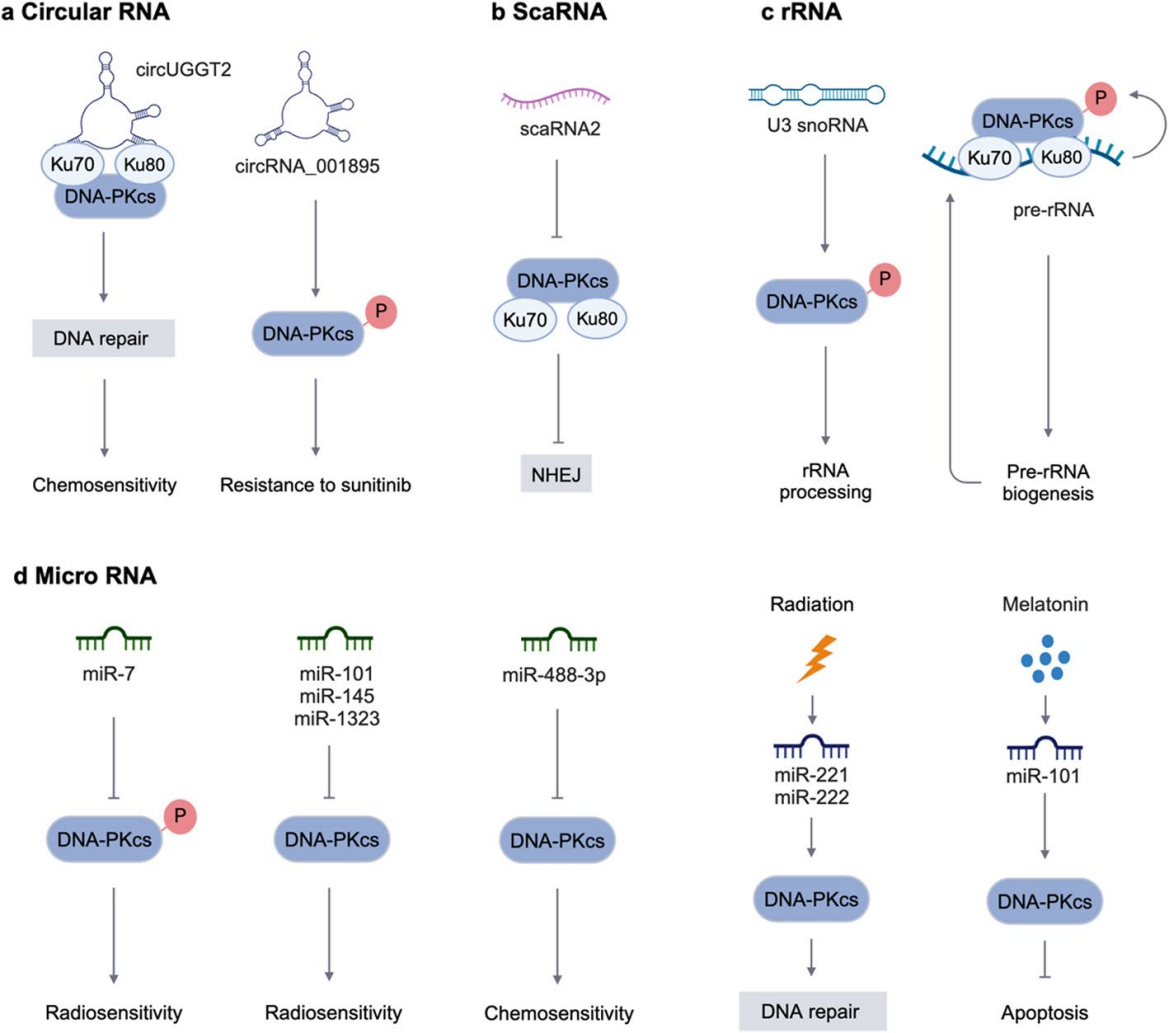
The interaction between DNA-PKcs and other noncoding RNAs. **a** The interaction between DNA-PKcs and circular RNAs. **b** ScaRNA2 weakens the interaction with Ku70/Ku80, thereby preventing the assembly and activation of the DNA-PK complex. **c** The roles of DNA-PKcs in rRNA processing and pre-rRNA biogenesis. **d** The interaction between DNA-PKcs and micro RNAs. The figure was created with BioRender.com. P, phosphorylation

CircUGGT2 combines with the KU heterodimer to recruit DNA-PKcs to damaged chromatin sites, thereby promoting H2AX phosphorylation and DSB repair [71]. CircRNA_001895 is elevated in sunitinib-resistant renal cell carcinoma (RCC). The silencing of CircRNA_001895 induced decreased levels of p-DNA-PK and Rad51 and increased levels of γH2AX [72]. This suggests a potential role for circRNAs in tumor drug resistance, but the mechanisms affecting DNA-PK phosphorylation need to be further investigated (Fig. 4a).

### MiRNAs

Unlike lncRNAs, most interactions between miRNAs and DNA-PKcs result in an impact on DDR repair in chemotherapy or radiotherapy. MiR-488-3p is significantly downregulated in clinical specimens and cell lines of malignant melanoma (MM) and its effect on the sensitivity of MM cells to cisplatin is mediated by DNA-PKcs. Overexpression of DNA-PKcs eliminates the promotion of miR-488-3p–induced sensitivity to cisplatin in MM cells [73]. MicroRNA-145 inhibits the translation of DNA-PKcs by binding to the 3′ untranslated region (UTR) of DNA-PKcs, resulting in accumulation of DNA damage [74]. Consistently, miR-1323 regulates the expression of DNA-PKcs protein by binding to the 3′-UTR of PRKDC, thereby participating in the radiation resistance of human lung cancer cells [75]. DSBs were induced by DNA-damage agents, then histone H2AX phosphorylation at Ser139 (γH2AX) increased. The level of γH2AX indicates the degree of DNA damage. Ectopic overexpression of miR-7 prolongs radiation-induced γH2AX lesion formation and reduces DNA-PKcs phosphorylation levels, thereby increasing the radiosensitivity of human cancer cells [76]. Similarly, ectopic overexpression of miR-101 effectively reduced ATM and DNA-PKcs levels in H1299, H1975, and A549 cells and increased radiosensitivity [77]. Moreover, research reported that melatonin upregulates DNA-PKcs by inhibiting miR-101 in HUVECs [78]. Genotoxic stress can also cause changes in miRNAs. Radiation induced transcription of miR-221 and miR-222. MiR-221 and miR-222 regulate DNA-PKcs expression by activating Akt, thereby affecting DNA damage repair [79]. These findings reveal the interaction network between noncoding RNA and DNA-PKcs (Fig. 4d).

### ScaRNA

Besides lncRNAs, miRNAs, and circRNAs, DNA-PK could also interact with scaRNA2. After binding to DNA-PKcs, scaRNA2 weakens its interaction with Ku70/Ku80, thereby preventing the assembly and activation of the DNA-PK complex [80] (Fig. 4b).

### Pre-rRNA biogenesis

In addition, DNA-PK plays an important role in ribosome biogenesis and may be a part of the pre-rRNA ribonucleoprotein complex. U3 snoRNA triggers Thr2609 site DNA-PKcs phosphorylation. Ku heterodimer mediates the catalytic deactivation or assembly of T2609 phosphorylation-defective DNA-PK, leading to rRNA processing defects [81].

Moreover, other researchers have found that DNA-PK may regulate pre-rRNA biogenesis in multiple steps, including pre-rRNA synthesis, modification, and processing. In addition, DNA-PK could recognize pre-rRNA. The absence of activated DNA-PK not only inhibits the transcription of 47S pre-rRNA but also suppresses the processing of the 18S rRNA precursor [82] (Fig. 4c).

## DNA-PKcs in alternative splicing

Alternative splicing (AS) is an important mechanism of transcriptional regulation that generates transcriptome diversity. DNA-PKcs are involved in pre-mRNA alternative splicing. A set of pre‐mRNAs in human lung adenocarcinoma A549 cell cells treated with the topoisomerase II inhibitor MTX was altered including SRSF1, SRSF2, HNRNPDL, HNRNPH1, and PRPF38B [9]. This supports the involvement of DNA-PKcs in controlling pre-mRNA splicing in response to MTX-induced genotoxic stress.

In addition, researchers found that knockdown and pharmacological inhibition of DNA-PKcs resulted in significant negative enrichment of the cell “spliceosome” gene set, indicating interference of splicing activity in cells with impaired DNA-PKcs activity. Further research showed DNA-PKcs regulated RNA binding motif protein X-linked (RBMX) transcription by binding to its *cis*-regulatory elements proximal to the transcriptional start site. The DNA-PKcs-RBMX regulatory loop controls AR transcript maturation and downstream AR isoform transcriptional activity by affecting splicing decisions of appropriately spliced exons 2 and 3 [83].

Our previous study showed the global gene landscape of DNA-PKcs-RNA interactions in human osteosarcoma U2OS cell and KEGG pathway analysis showed that many RNAs were involved in cell adhesion and receptor ECM interaction pathways. In addition, we have demonstrated that DNA-PKcs affects CD44 alternative splicing, and the expression of CD44 variants increases after DNA-PK inhibition [84]. This provides insights into how alternative splicing can promote DNA-PK mediated DDR responses.

## Perspectives and conclusions

Although many studies have reported the non-DDR function of DNA-PKcs, there are still several molecular mechanisms to be explored [85].

### The intersection of classical and nonclassical functions of DNA-PKcs

As mentioned earlier, DNA-PK is involved in the biogenesis of rRNA without genotoxic stress. Simultaneous treatment of low-dose DNA-PK-i and PARP-i will induce synthetic lethality in BRCA1 deficient triple negative breast cancer cell lines, which is caused by defects in pre-rRNA biogenesis rather than DNA damage reaction. Interestingly, DNA-PK exhibited the opposite function after genotoxic stress. DNA-PK acts upstream of PARP-1 and inhibits rRNA synthesis after DNA damage. DNA-PK and PARP-1 inhibitors prevented cisplatin induced rRNA synthesis blockade [86].

The nucleolus is located in the core of the nucleus and occupies a large amount of space in the nucleus. Its main function is to produce and assemble ribosome complexes [87, 88]. Gamma H2AX is a substitute marker for DSB and a substrate for DNA-PKcs, mainly present in the nucleoplasm and not in the nucleolus. Studies have found that DNA-PKcs exists in the nucleolus even in the absence of genetic stress toxicity [82]. This indicates a correlation between the subcellular localization and function of DNA-PKcs. However, the regulatory mechanism is not clear. Ku heterodimer and pre-rRNA may have some functions; it is worth further exploration.

### DNA-PKcs in the intersection of DNA damage repair and transcription

DNA lesions from both exogenous and endogenous-derived insults will directly or indirectly impede the transcription. The transcription-coupled DNA repair (TCR) pathway is crucial to eliminate such DNA lesions. When DSB occurs within or near actively transcribed genes, DNA-PK and HECT E3 ubiquitin ligase WWP2 are recruited to the damage sites, leading to ubiquitination of RNAPII subunit RPB1 and triggering RNAPII degradation, thereby promoting transcriptional silencing of broken genes [22]. However, it is unknown if DNA-PKcs functions in the TCR machinery or in the repair of transcription-induced DNA breaks or in the resolution of transcription-replication conflicts (TRCs) [89].

### Ku70/Ku80 cooperation with DNA-PKcs in DDR and RNA metabolism

Ku70/Ku80, as a part of the DNA-PK complex, also plays an important role in DDR and RNA metabolism. Ku70/Ku80 recognizes and binds to the end of DNA, and the kinase activity of DNA-PKcs is fully activated only in the presence of DNA and Ku70/Ku80 [90, 91]. The N-terminal α-solenoid of DNA-PKcs consists of an N-terminal and middle Huntingtin, elongation factor 3, PP2A, and TOR1 (HEAT) repeats (N-HEAT and M-HEAT) [92]. N-HEAT is divided into two fragments (NH1 and NH2), while M-HEAT is divided into three fragments (MH1, MH2, and MH3). The cryoelectron microscopy structures of DNA-PK reveals that Ku stabilizes the DNA-PKcs DNA complex by prolonging DNA binding, causing DNA-PKcs to switch to an activated state. Moreover, Ku contacts NH1 of N-HEAT and MH3 of M-HEAT, bridging both sides of the DNA binding slot in DNA-PKcs and fixing N-HEAT of DNA-PKcs onto DNA [93]. Ku binds to substrates to promote DNA-PKcs phosphorylation of substrates. In vitro experiments have shown that the binding of Oct-1 to Ku promotes DNA-PK phosphorylation of Oct-1 [94]. In addition, the Ku protein can also affect the nonclassical functions of DNA-PKcs. Ku drives the assembly of DNA-PKcs on RNA, promoting the biogenesis of ribosomes. Interestingly, Li et al. also demonstrated that DNA-PK regulates rRNA biogenesis by phosphorylating ribosomal proteins. In addition, they found that pre-rRNA is a functional partner of the Ku70/Ku80 complex in the nucleolus. In addition, the Ku subunit is necessary for targeting DNA-PKcs to appropriate positions in the nucleolus. Therefore, they proposed a positive feedback hypothesis between Ku70/Ku80 complex, pre-rRNA, and DNA-PKcs. The interaction between Ku complex and pre-rRNA may mediate the activation of DNA-PKcs in the nucleolus [82].

### The prospects of DNA-PK targeting based therapy

Numerous studies have focused on the expression of DNA-PKcs in tumor cells [5, 95–97]. DNA-PKcs is involved in multiple cellular processes during tumor occurrence and progression, including the cell cycle, proliferation, metastasis, and transcriptional regulation [2]. DNA-PKcs is dysregulated in a variety of tumors, including prostate, breast, and cervical cancers. Genetic or pharmacological approaches have been used to reduce DNA-PK expression in tumor cells, thereby increasing their sensitivity to radiation or chemotherapy agents. The therapeutic effect of DNA-PKcs inhibitors in tumors may be due to their inhibition of the activity of certain transcription factors [4, 7, 11]. Given the oncogenic effect of DNA-PK, inhibitors targeting DNA-PK kinase activity have been developed and tested in clinical trials [4]. Although the in vitro results of the DNA-PK inhibitor experiments were encouraging, the in vivo research results were unsatisfactory, possibly owing to the limited solubility of NU7441 in vivo, which leads to poor absorption in mice. Compared with NU7441, targeting DNA-PK with a clinical-grade dual inhibitor of DNA-PK and TOR kinase (TORK) (CC-115) inhibits the proliferation of CRPC cells and affects DNA-PKcs dependent transcriptional events, including cancer-related pathways such as epithelial-mesenchymal transition and transforming growth factor β (TGFβ) signalling [4].

Though DNA-PKcs is extensively studied in tumors, the specific function of DNA-PKcs in immune cells remains to be elucidated. Owing to the resolution of the tumor microenvironment, cell therapy and immunotherapy play an increasingly important role in the field of precision cancer treatment [98]. DNA-PKcs, a key point in T cell activation, could promote Egr1 transcription factor dependent IL2 transcription [49]. However, combination therapy with regorafenib and NU7441 increased the activation of T cells and co-stimulatory markers as well as cytokines such as IFNγ, TNFα, and IL2 [99]. These studies suggest that the pharmacological inhibition of DNA-PKcs may serve as a method to enhance T cell immunotherapy.

The combination therapy of DNA-PK inhibition and mRNA splicing is a promising strategy. DNA-PKcs could regulate mRNA splicing, and splicing activity is impaired in cells with reduced DNA-PKcs activity. To identify genes that promote DNA-PK inhibitors resistance, researchers conducted CRISPR/Cas9 screening and identified genes related to RNA splicing. Moreover, pladienolide B-mediated splicing inhibition enhances the cytotoxicity of DNA-PK inhibitors in various human cancer cell types. This indicates that the combination therapy of DNA-PK inhibitors and mRNA splicing inhibitors has the potential for tumor treatment in vitro. However, the mechanisms of combination therapy and how RNA splicing related genes promote DNA-PK inhibitors resistance are still unclear.

In conclusion, targeting DNA-PKcs is a promising therapeutic strategy that can produce significant anticancer effects. Further understanding of the functions of DNA-PKcs, particularly transcriptional regulation and RNA metabolism, could provide insights for developing effective therapeutic approaches.

## Abbreviations

DNA-PKcs: DNA-dependent protein kinase catalytic subunit
PIKK: Phosphatidylinositol 3-kinase-related kinase
NHEJ: Nonhomologous DNA end-joining
lncRNAs: Long noncoding RNAs
miRNAs: Micro RNAs
circRNAs: Circular RNAs
Lama4: Laminin alpha 4
Nrf2: NF-E2-related factor 2
hXOR: Human xanthine oxidoreductase gene
OSM: Oncostatin M
TopoIIβ: DNA topoisomerase IIβ
PARP-1: Poly [adenosine diphosphate (ADP)–ribose] polymerase–1
Pol II: RNA polymerase II
DSB: DNA double-strand break
HIFs: Hypoxia-inducible factors
TRIM28: Tripartite motif containing protein 28
HREs: Hypoxia response elements
CDK9: Cyclin-dependent kinase 9
CTD: C-terminal domain
FoxA2: Forkhead box A2
CRY: Cryptochrome
PDX-1: Pancreatic duodenal homeobox-1 protein
EMT: Epithelial-mesenchymal transition
GSCs: Glioma stem cells
HSF1: Heat shock transcription factor 1
PR: Progesterone receptor
AIRE: Autoimmune regulatory factor
mTECs: Myeloid thymic epithelial cells
TLR: Toll-like receptor
Egr1: Early growth response protein 1
IRF-3: IFN regulatory factor 3
FAS: Fatty acid synthase
WDR6: WD40 repeat-containing protein 6
USF-1: Upstream stimulatory factor 1
Zic2: Zinc finger protein of cerebellum 2
RHA: RNA helicase A
NF-κB: Nuclear factor-kappa B
NEMO: NF-κB essential modulator
EGFR: Epidermal growth factor receptor
MYT1L: Myelin transcription factor 1-like
LEF1: Lymphoid enhancer binding factor 1
CRPC: Castration-resistant prostate cancer
DDR: DNA damage repair
YB-1: Y-box binding protein
BCLAF1: Bcl-2-associated transcription factor 1
KLF8: Krüppel-like factor 8
NR4A: Nuclear receptor subfamily 4 group A
SRF: Serum response factor
MTX: Methotrexate
AS: Alternative splicing
RBMX: RNA binding motif protein X-linked
HEAT: Huntingtin, elongation factor 3, PP2A, and TOR1
TCR: T cell receptor; transcription-coupled repair
